# Synaptive Magnetic Resonance Imaging for Stereotactic Radiosurgery

**DOI:** 10.7759/cureus.75600

**Published:** 2024-12-12

**Authors:** Michael Chaga, Timothy Chen, Wenzheng Feng, Darra Conti, Jing Feng, Tingyu Wang, Ma Rhudelyn Rodrigo, Elizabeth Luick, Daniel Thompson, Joy Baldwin, Brielle Latif, Joseph Hanley, Shabbar Danish

**Affiliations:** 1 Radiation Oncology, Jersey Shore University Medical Center, Neptune, USA; 2 Neurological Surgery, Jersey Shore Medical Center, Neptune, USA

**Keywords:** magnetic resonance imaging, mri, radiosurgery, srs, stereotactic radiosurgery, synaptive

## Abstract

Introduction

The Synaptive magnetic resonance imaging (MRI) system (Synaptive Medical, Toronto, Canada) is a midfield 0.5 T head-only scanner for imaging the head and neck in adults and pediatrics. The system received US FDA and Health Canada clearance for clinical use in 2020. Initial installations occurred at sites throughout Canada with the first international installation occurring in the USA at Hackensack Meridian’s Jersey Shore University Medical Center (JSUMC) in October 2023. The design of the Synaptive MRI allows the system to be installed and operated outside the context of standard Radiology facilities. In this study, we describe the implementation and adaptation of the Synaptive MRI into the cranial stereotactic radiosurgery (SRS) workflow, with the intention of reducing the time between consultation, MRI, and treatment.

Methods

The Synaptive MRI was installed next to the cranial SRS suite in the Radiation Oncology (RO) department, dedicated solely to SRS planning image acquisition. Geometric distortion was evaluated using the Magphan 128 Distortion Phantom (Phantom Laboratory, Greenwich, NY). The simplicity of the Synaptive interface allows Radiation Therapist operation, in the State of New Jersey, without the need for the physical presence of an MRI technologist. During imaging acquisition, the Physician verifies image quality and can rescan or make adjustments as needed, allowing for instantaneous confirmation of image quality. The Synaptive comes with a full set of neuro pulse sequences and protocols ranging from T1 3D spoiled gradient recalled echo to time-of-flight magnetic resonance angiography. Patient MRI comfort was evaluated after treatment by questionnaire for 51 patients using a Likert scale from 1 to 5 (1 = “very poor”, 5 = “very good”). The total Synaptive MRI time for 38 patients was tracked from arrival at the MRI suite to completion of imaging. Times from consult, imaging, and treatment for 58 ZAP-X (ZAP Surgical Systems, Inc., San Carlos, CA) SRS patients at JSUMC RO in 2024 who received Synaptive MRI were obtained from Aria (Varian Medical Systems, Palo Alto, CA) electronic medical records. JSUMC RO Departmental data was obtained on times from consult, imaging, and treatment for 58 randomly sampled patients from 2018 to 2023 whose treatment planning imaging was performed on out-of-department MRI units for comparison.

Results

The distortions on this system are less than 0.52 mm, for distances up to 90 mm from the isocenter. The average MRI comfort was 4.7 ± 0.5. The average total MRI time for 38 patients was 36 ± 3 minutes. The median time for 58 ZAP-X SRS patients from MRI to treatment was seven days (interquartile range: p25 = six days, p75 = 10 days), consult to MRI 1.5 days (p25 = 0 days, p75 = nine days), and consult to treatment 12 days (p25 = seven days, p75 = 19 days). The median time for 34 malignant ZAP-X SRS patients from MRI to treatment was six days (p25 = five days, p75 = eight days) and from consult to treatment nine days (p25 = six days, p75 = 18 days). Significant decreases in MRI to treatment times (P < 0.001) and benign consult to MRI times (P = 0.0039) were demonstrated for 2024 Synaptive patients compared to 2018-2023 patients.

Conclusion

Dedicated Synaptive MRI installation for SRS in RO departments can improve the SRS workflow, potentially providing a more streamlined experience and reducing the time from diagnosis and imaging to treatment for cranial pathologies.

## Introduction

The Synaptive magnetic resonance imaging (MRI) system (Synaptive Medical, Toronto, Canada) is a midfield 0.5 T scanner for imaging the head and neck in adults and pediatrics. The system received US FDA and Health Canada clearance for clinical use in 2020. Initial installations occurred at sites throughout Canada with the first international installation occurring in the USA at Hackensack Meridian’s Jersey Shore University Medical Center (JSUMC) in October 2023 [1]. The design of Synaptive MRI allows the system to be installed and operated outside the context of a standard Radiology department. Our purpose was to utilize the system to acquire planning images for a dedicated cranial stereotactic radiosurgery (SRS) program. There are no reports describing the use of the Synaptive MRI in this context. In this study, we describe the implementation and adaptation of the Synaptive MRI into the cranial SRS workflow.

## Materials and methods

Overview and installation

**Figure 1:**
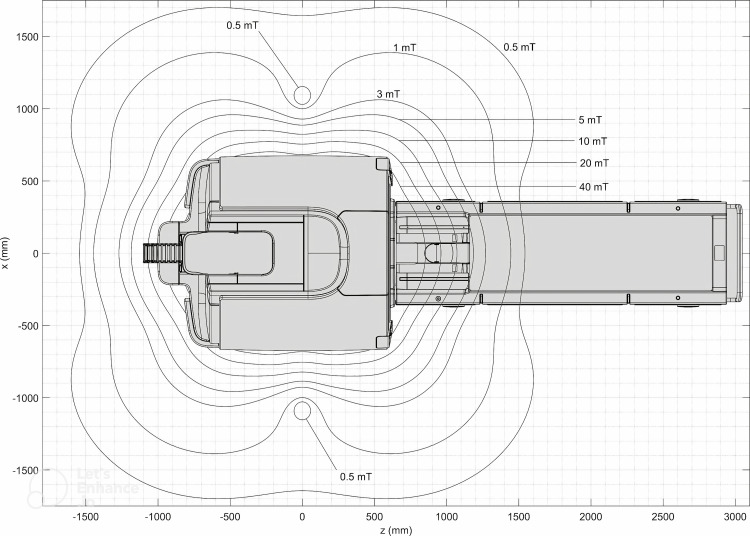
Synaptive MRI fringe field contours, top-view. Image credit: Synaptive Medical, used with permission

**Figure 2:**
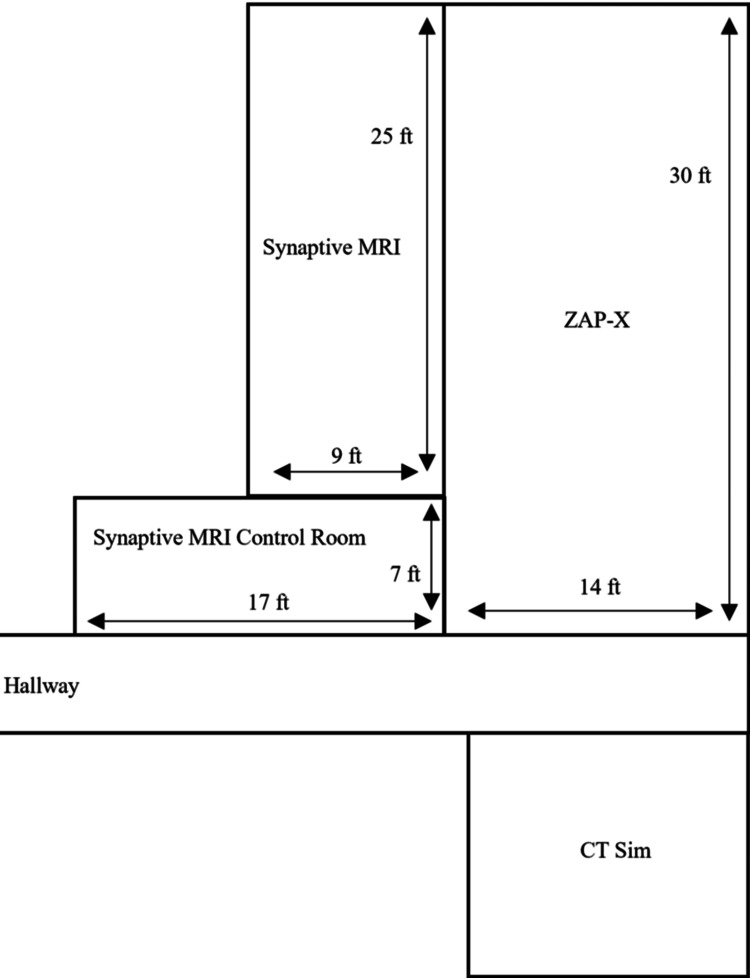
JSUMC RO department layout demonstrating co-location of Synaptive MRI with ZAP-X SRS and CT simulation. Image credit: Michael Chaga. Not drawn to scale. JSUMC: Jersey Shore University Medical Center, RO: Radiation Oncology, SRS: stereotactic radiosurgery, CT: Computed Tomography

The Synaptive MRI system comprises a 0.5 T cryogen-free superconducting magnet, a high-performance gradient coil, and an advanced radiofrequency system. The cryogen-free magnet design allows the user to magnetize and demagnetize the system at will and does not require a quench pipe. The gradient subsystem operates at a maximum amplitude of 100 mT∙m^-1^ and a maximum per-axis slew rate of 400 T∙m^-1^∙s^-1^. The radiofrequency transmit system has a peak output of 60 μT. The scanner produces a maximum acoustic output of 99 dB(A) in the scanner room. The Synaptive magnet is less than 1,900 lbs and can be installed in under 250 ft^2^, with the 0.5 mT line approximately 5 ft from the center of the bore (Figure 1) [1-3]. The small footprint and cryogen-free superconducting magnet allows the system to be installed in the Radiation Oncology (RO) Department. The Synaptive MRI suite was installed in close proximity to the ZAP-X (ZAP Surgical Systems, Inc., San Carlos, CA) SRS and computed tomography (CT) simulation areas (Figure 2). The room housing the Synaptive is 9 ft × 25 ft including an equipment area. The Synaptive control room is 17 ft × 7 ft. The MRI suite was designed with typical magnetic and radiofrequency shielding to minimize interference with surrounding clinical equipment and for direct observation of the patient via a radiofrequency-shielded window [4].

Quality assurance 

For intracranial SRS, which depends on highly accurate image guidance, planning is generally based on MRI because of its soft tissue contrast and sensitivity for delineating radiation targets and normal brain tissue. However, MRI is subject to anatomic distortion from multiple sources, including static-field inhomogeneity, eddy currents, and gradient field nonlinearity which may lead to missed targets and unnecessary treatment of normal brain tissue if uncorrected [5]. To assess the magnitude of distortion, a geometric accuracy test was performed with the Magphan 128 (EMR128) Distortion Phantom (Phantom Laboratory, Greenwich, NY) [6]. Three scans were taken with 1 mm isotropic resolution 3D gradient echo sequence using axial RL (read along x), axial AP (read along y), and sagittal HF (read along z) over a 220 mm field of view (220 × 220 × 220 matrix size) and 64 kHz bandwidth (267 Hz∙pixel^-1^). The distortion value (DV) was obtained with the Smári image analysis service (Phantom Laboratory, Greenwich, NY) [7]. For each scan, a comma-separated values file is generated containing the designed (ideal) x, y, and z positions of the markers and the measured x, y, and z positions of the markers. Calculating the vector distance between these points gives the DV at each marker location. Distortion along the readout direction of any given scan will be slightly influenced by the background magnetic field homogeneity B_0;_ however, due to the relatively high readout bandwidth of these scans, this effect should be minimal [8,9]. Prior to patient imaging, daily quality assurance tests are performed such as functional, central frequency, transmit gain, signal-to-noise, and spatial fidelity checks (Table 1).

**Table 1:** Synaptive MRI daily quality assurance.

Procedure	Tolerance
Safety	
Intercom/viewing window	Functional
Emergency squeeze ball	Functional
Emergency couch release	Functional
Safety signage	Functional
Bore clearance	Functional
Metal detector	Functional
Battery charged	Functional
Imaging	
Central frequency	21.273 – 21.277 MHz
Transmit gain/B1 maximum	69.2 – 76.4 μT
Signal-to-noise	> 100
Spatial fidelity x and y	155 ± 2 mm

Workflow

The Synaptive MRI is dedicated solely to SRS at JSUMC RO. After RO consultation and insurance authorization, patients are scheduled for a same-day CT Sim and MRI with a goal of ≤ 5 days between consult and imaging for benign cases and same-day consult and imaging for malignant cases. Following thermoplastic mask creation and CT Sim, the SRS patient is immediately escorted across the hall to receive their MRI scan. The intravenous line is established by the RO Nurse for the gadolinium contrast agent if applicable. The patient is positioned on the machine in the head coil by the Nurse and Radiation Therapist where only their head and neck are enclosed. Contrast is delivered through an automated injector with concentration 0.2 mL∙kg^-1^ with 1 mL∙sec^-1^ and flush 15 mL with 1 mL∙sec^-1^. The time interval between contrast injection and the start of contrast-enhanced image acquisition is five minutes. Patient communication is maintained through a speaker and microphone system built into the patient positioner in the scanner and an alarm squeeze bulb. The simplicity of the Synaptive interface with preset imaging parameters and push-button automated scanning permits Radiation Therapist operation in the State of New Jersey without the physical presence of an MRI technologist. This allows the machine to sit outside the Radiology Department. During imaging acquisition, the Physician verifies image quality and can rescan or make adjustments as needed, allowing for instantaneous confirmation of image quality. After imaging, patients are scheduled for SRS treatment with a goal of ≤ 5 days between imaging and treatment. Contraindications for imaging on Synaptive at JSUMC RO include the presence of ferromagnetic implants or non-MR-compatible devices, severe claustrophobia, or inability to hold the immobilized position for the entire scan duration. 

Imaging protocols and radiosurgical planning

For imaging acquisition, the Synaptive comes with a full set of neuro pulse sequences and protocols ranging from T1 3D Spoiled gradient recalled echo to time-of-flight magnetic resonance angiography. Table 3 demonstrates protocols currently used for SRS planning and their parameters. Images are automatically sent to Synapse PACS and Eclipse (Varian Medical Systems, Palo Alto, CA) via the Synaptive DICOM Networking Interface. CT and MRI images are registered in Eclipse for contouring and imported to ZAP-X TPS for SRS planning. 

**Table 2:** Synaptive MRI scanning protocols used for SRS planning at JSUMC RO department. SPGR: Spoiled Gradient Recalled Echo, SSFP: Steady State Free Precession, FLAIR: Fluid Attenuated Inversion Recovery, TOF-MRA: Time-of-Flight – Magnetic Resonance Angiography, GBM: Glioblastoma, Pituitary: Pituitary Adenoma, TN: Trigeminal Neuralgia, AVM: Arteriovenous Malformation, VS: Vestibular Schwannoma, Spinal: Spinal Tumor

Protocol	Repetition time (ms)	Echo time (ms)	Flip angle (°)	Slice thickness (mm)	Field of view (cm)	Scan time (s)	Pathologies
T1 3D SPGR	11.04	5.16	23	0.56	25	322	Brain mets, GBM, meningioma, pituitary, TN, AVM, hemangioma, VS, spinal
SSFP 3D 0.8 mm	6.78	3.33	35	0.40	25	276	Brain mets, GBM, meningioma, pituitary, TN, AVM, hemangioma, VS
FLAIR	7132.13	110.31	90	4	24	244	Brain mets, GBM
T1 3D SPGR delayed	11.04	5.16	23	0.56	25	322	Brain mets, GBM, VS
T2	6,327.27	102.62	90	5	24	259	Meningioma, AVM, hemangioma, VS
TOF-MRA	21.70	5.66	30	0.50	20	295	AVM, hemangioma

Comfort and time evaluation

Patient MRI comfort was evaluated after treatment by questionnaire for 51 patients using a Likert scale from 1 to 5 (1 = “very poor”, 5 = “very good”). The total Synaptive MRI time for 38 patients was tracked from arrival at the MRI suite to completion of imaging. Times from consult, MRI, and treatment for 58 ZAP-X SRS patients at JSUMC RO in 2024 who received Synaptive MRI were obtained from Aria (Varian Medical Systems, Palo Alto, CA) electronic medical records. JSUMC RO Departmental data were obtained on times from consult, MRI, and treatment for 58 randomly sampled patients, treated utilizing Linear Accelerator (Linac)-based SRS from 2018 to 2023 for comparison with Synaptive MRI patients. The treatment planning imaging for these patients was performed on out-of-department MRI units.

Statistical analysis 

Statistical analysis was performed with Stata 18 (Release 18. StataCorp LLC, College Station, TX) using mean, median, standard deviation, interquartile range, minimum, maximum, Shapiro-Wilk, Shapiro-Francia, skewness and kurtosis normality tests, and two-sample Wilcoxon rank-sum (Mann-Whitney U) test. Statistical significance was defined as a P-value less than 0.05.

## Results

**Figure 3:**
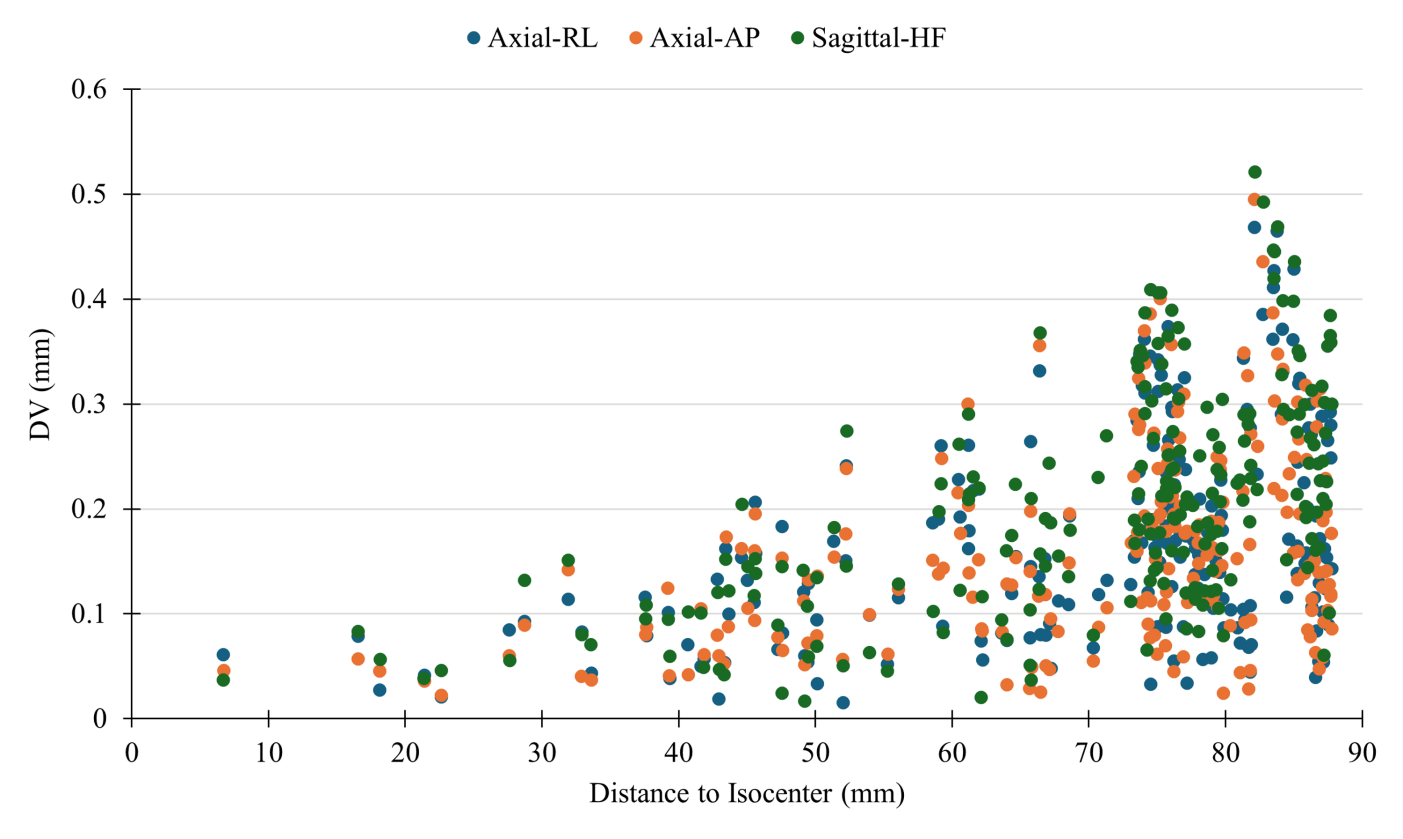
DV as a function of distance from isocenter for each of the three scans used to analyze Synaptive MRI geometric distortion. Image credit: Michael Chaga DV: distortion value

**Figure 4:**
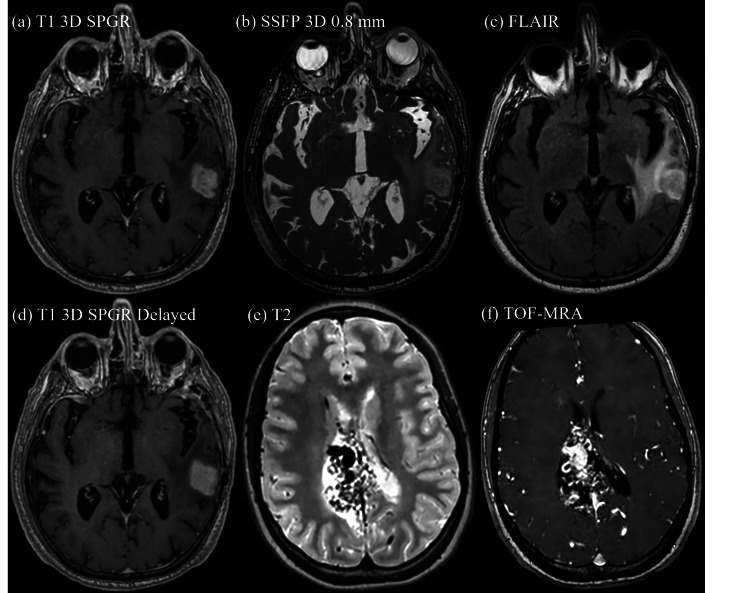
Representative images for JSUMC Synaptive MRI protocols: (a–d) Posterior lateral temporal lobe glioblastoma. (e–f) Right occipital lobe arteriovenous malformation. Image credit: Michael Chaga JSUMC: Jersey Shore University Medical Center

The average DV for axial RL was 0.2 ± 0.1 mm, for axial AP 0.16 ± 0.09 mm, and for sagittal HF 0.2 ± 0.1 mm (Table 3). This is within the recommended 2 mm DV tolerance [9-11]. DV as a function of distance from isocenter is given in Figure 3 for each scan, highlighting the effect of increasing distortion with respect to increasing radial distance from the scanner isocenter. From the data, the distortions on this system are less than 0.52 mm, for distances up to 90 mm from isocenter. The average MRI comfort was 4.7 ± 0.5. The average total MRI time was 36 ± 3 minutes. Table 4 shows the median times from consult, MRI, and treatment. The median time for 58 ZAP-X SRS patients at JSUMC RO in 2024 from MRI to treatment was seven days (interquartile range: p25 = six days, p75 = 10 days), from consult to MRI 1.5 days (p25 = 0 days, p75 = nine days), and from consult to treatment 12 days (p25 = seven days, p75 = 19 days). Significant decreases were demonstrated in MRI to treatment times (P < 0.001) and benign consult to MRI time (P = 0.0039) for 2024 Synaptive patients compared to 2018-2023 patients. The majority of 2018-2023 patients received their planning MRI before the RO consult, compared to Synaptive patients who received it after the consult. Representative images for each scanning protocol are shown in Figures 4a-4f.

**Table 3:** Average, range, and median DVs for each of the three scans used to analyze Synaptive MRI geometric distortion. †median (interquartile range: p25, p75)

Scan	Average (mm)	Range (mm)	Median (mm)†
Axial RL	0.2 ± 0.1	0.02 – 0.47	0.16 (0.09, 0.23)
Axial AP	0.16 ± 0.09	0.02 – 0.50	0.15 (0.09, 0.21)
Sagittal HF	0.2 ± 0.1	0.02 – 0.52	0.20 (0.12, 0.27)

**Table 4:** Median times from consult, MRI, and treatment at JSUMC RO department for 2024 ZAP-X SRS patients who received Synaptive MRI and 2018–2023 Linac-based SRS patients whose treatment planning imaging was performed on out-of-department MRI units. †median (interquartile range: p25, p75), two-sample Wilcoxon rank-sum (Mann-Whitney U) test, z-value, P < 0.05 significance

Pathology	Year	MRI to treatment (days)†			Consult to MRI (days)†			Consult to treatment (days)†		
All	2024 (n = 58)	7 (6, 10)	z = 5.131	P < 0.001	1.5 (0, 9)	z = 3.092	P = 0.0020	12 (7, 19)	z = -0.224	P = 0.8228
	2018 – 2023 (n = 58)	12 (9, 25)			5.5 (2, 16)			11 (7, 19)		
Malignant	2024 (n = 34)	6 (5, 8)	z = 4.541	P < 0.001	0 (0, 7)	z = 1.778	P = 0.0754	9 (6, 18)	z = -0.474	P = 0.6357
	2018 – 2023 (n = 34)	11 (8, 14)			4 (1, 7)			9 (7, 12)		
Benign	2024 (n = 24)	10 (7, 12)	z = 3.372	P < 0.001	5.5 (0, 14)	z = 2.887	P = 0.0039	14.5 (10, 24.5)	z = 0.815	P = 0.4148
	2018 – 2023 (n = 24)	25.5 (10, 86.5)			15 (6.5, 25.5)			19.5 (11.5, 30.5)		

## Discussion

For SRS patients, Synaptive MRI improves accessibility since it can be easily installed in RO departments, unlike other high-end traditional MRI modalities which require a large room to contain the magnetic field, a quench vent, acoustic noise considerations that restrict installation locations within hospitals and clinics, and dedicated Radiology personnel [1-3,12]. A dedicated SRS MRI scanner in RO departments allows for an easier workflow and can reduce the time from consult and MRI to treatment. Delays in treatment can occur because of institutional constraints, such as a limited number of MRI scanners or treatment appointments and the availability of staff with expertise in SRS simulation, planning, or delivery. The time taken for insurance approval for imaging or treatment affects scheduling and patient factors such as distance to treatment facility or illness may cause further delays to treatment [13]. Following imaging, SRS quality assurance measures are performed which typically result in delays on the order of five to 10 days [14]. Having a dedicated imaging acquisition system in RO departments can help prevent delays in treatment by simplifying the SRS workflow process. In a meta-analysis of four studies of head and neck cancer, Chen et al. found a significant decrease in survival with increasing delay time [15]. Seymour et al. performed a study to evaluate workflow and patient outcomes related to frameless SRS for brain metastases and found that local freedom from progression was lower with a median of six days from MRI to treatment and 13 days from consult to treatment [16]. Salkeld et al. performed a study to evaluate changes in brain metastases or resection cavity volumes between planning MRI and SRS treatment and found that 41% required a change in management with ≤ 7 days and 78% with ≥ 8 days [13]. The median time for 34 malignant ZAP-X SRS patients at JSUMC RO from MRI to treatment was six days (p25 = five days, p75 = eight days) and from consult to treatment nine days (p25 = six days, p75 = 18 days) as shown in Table 4. Compared to other high-end traditional MRI modalities for SRS, additional benefits of Synaptive MRI include less equipment, installation, and service costs, no helium refill, non-technical staff operation, and reduced chemical shift artifacts [1-3,8,12]. For SRS planning, lower magnetic field strength MRI systems such as Synaptive are superior to traditional higher field strength systems due to decreased magnetic susceptibility, leading to decreased geometric distortion in the image and positional errors during treatment [17].

Several limitations should be considered in this study on analyzing Synaptive MRI for SRS. The study was conducted at a single institution, limiting the generalizability of findings. Other institutions with different patient populations, operational setups, and equipment may encounter varied results. The sample size, although reflective of the patient volume at our institution, is modest. A larger and more diverse sample size would provide robust data and enhance the strength of the conclusions. The comfort and time evaluations are subjected to selection bias. Additional long-term data would be necessary to determine the impact on clinical outcomes or overall patient survival from incorporating Synaptive MRI for SRS.

## Conclusions

The Synaptive’s attributes are ideal to create a streamlined SRS program. Dedicated Synaptive MRI installation for SRS in RO departments can improve SRS workflow and eliminate hurdles that exist to reduce the time from diagnosis and imaging to SRS treatment for cranial pathologies.
